# PReS-FINAL-2198: Insufficient efficacy of tocilizumab therapy in children with Castleman's disease

**DOI:** 10.1186/1546-0096-11-S2-P188

**Published:** 2013-12-05

**Authors:** A Kozlova, D Abramov, N Miakova, A Maschan, A Shcherbina

**Affiliations:** 1 Immunology, Federal Research and Clinical Center of Pediatric Hematology, Oncology and Immunology, Moscow, Russian Federation

## Introduction

Castleman's disease (CD) or angiolymphoid vascular hyperplasia is a rare and not well recognized disease of unknown etiology characterized by a massive growth of lymphoid tissue that was first described in 1954, and subsequently better defined by Castleman in 1956. It usually appears in young adults whilst it rarely occurs in childhood. There are only about 100 pediatric cases published. CD is believed to be autoinflammatory in pathology and result in increased interleukin-6 secretion. Previous studies in adult patients suggested beneficial role of the anti-interleukin-6 receptor antibody tocilizumab in treatment of CD. We report 3 cases of multicentric Castleman's disease in children, treated in our center in recent years.

## Objectives

To assess efficacy of tocilizumab therapy in children with Castleman's disease.

## Methods

3 patients ages 4, 14 and 16 were diagnosed with multicentric Castleman's disease on the basis of clinical and histologic findings. 2 children (the boy of 4 years and the girl of 14 years) had hyaline-vascular type of CD, the boy of 16 years had plasma cell type of CD. The duration of illness before the correct diagnosis and beginning of treatment in the younger boy was 6 months, in the girl and the older boy - 6 years. All patients had enlarged lymph nodes of several groups and systemic involvement with fevers, weight loss, ascites, and progressive hematological changes (thrombocytopenia, anemia, high CRP). HHV-7 and 8 staining of the tissues were negative. In all cases treatment with tocilizumab as monotherapy was started at a dose of 8 mg/kg every 2 weeks.

## Results

2 infusions in 2 children and 1 in one was performed. During the treatment period lymph nodes' sizes slightly decreased two patients and no lymph nodes size change was noted in the third patient. All patients continued to have progression of systemic symptoms. Therefore, Tocilizumab treatment was discontinued and substituted with more aggressive protocols consisting of cyclophosphamide, vincristine, prednisone and rituximab with fast effect: two children now are disease-free, the third one has marked improvement.

## Conclusion

Tocilizumab has been reported to be effective for treatment of adults with CD, yet it proved insufficient activity in our three pediatric cases. In our experience CD in children has more aggressive course, therefore requiring chemotherapy aimed at lymphocytes' reduction.

## Disclosure of interest

None declared.

